# Machine learning prediction of physician-assigned treatment categories from pre-procedural electronic medical record data in cardiac patients: A multiclass classification study

**DOI:** 10.1371/journal.pdig.0001149

**Published:** 2026-07-16

**Authors:** Mohammad Tanhaei

**Affiliations:** Department of Engineering, Ilam University, Ilam, Iran; Liverpool John Moores University - City Campus: Liverpool John Moores University, UNITED KINGDOM OF GREAT BRITAIN AND NORTHERN IRELAND

## Abstract

Cardiovascular diseases (CVDs) remain a leading source of morbidity, mortality, and healthcare burden worldwide. In patients with coronary artery disease (CAD), choosing among medical therapy, percutaneous coronary intervention (PCI), and coronary artery bypass grafting (CABG) is clinically complex and depends on multiple pre-procedural factors. In this retrospective multicenter registry study, we developed a multiclass machine learning model to predict *physician-assigned treatment categories* rather than clinically optimal treatment. The analytic cohort included 2,682 unique patient records from the BioArc Clinical Registry, with class imbalance across medical therapy (*n* = 1,634), PCI (*n* = 817), and surgery (*n* = 231). Semi-structured JSON records were parsed to extract pre-decision demographic, clinical, symptom-related, and risk-factor variables. Missing data handling, encoding, SMOTE, recursive feature elimination (RFE), and hyperparameter tuning were performed within a nested stratified cross-validation workflow to reduce dependence on a single data split and to limit information leakage. The final XGBoost workflow retained **18** predictors after RFE. Across the outer folds of nested cross-validation, the model achieved an overall accuracy of **82.3% ± 1.5%** (95% CI: **80.8%–83.8%**) and a macro F1-score of **0.775 ± 0.018** (95% CI: **0.757–0.793**), with one-vs-rest AUC values of **0.78 ± 0.02**, **0.75 ± 0.02**, and **0.80 ± 0.03** for medical therapy, PCI, and surgery, respectively. Class-specific performance and confusion patterns were summarized from outer-fold predictions. Model interpretation using SHAP and partial dependence plots identified age, systolic blood pressure, symptom-related features, and opium consumption as influential predictors in this dataset. These findings indicate that pre-procedural EMR data contain signal related to observed treatment-assignment patterns; however, the model does not identify the optimal treatment or demonstrate improved clinical outcomes. External validation, external calibration, and prospective outcome-based evaluation are required before clinical deployment.

## Author summary

Choosing between medical therapy, PCI, and CABG in patients with coronary artery disease is often challenging, especially before invasive testing. We used a cardiovascular registry containing pre-procedural clinical information to train a machine learning model that predicts which treatment category physicians selected in routine care. The model was evaluated using nested cross-validation rather than a single train–test split, and feature selection was performed inside the validation workflow. Class-specific performance and confusion patterns were evaluated, showing strong separation for the surgery class and greater overlap between medical therapy and PCI. Age, blood pressure, symptom-related features, and opium consumption were among the most influential predictors in this dataset. These findings show that pre-procedural electronic medical record data contain useful signal about observed treatment-assignment patterns. However, the model does not identify the optimal treatment for a patient and should not yet be used as a prescriptive clinical tool. Further external validation and outcome-based studies are needed before implementation.

## 1 Introduction

Cardiovascular diseases (CVDs) remain a major cause of mortality and healthcare burden worldwide [1]. In patients with coronary artery disease (CAD), treatment decisions commonly involve medical therapy, percutaneous coronary intervention (PCI), or coronary artery bypass grafting (CABG). These decisions are informed by clinical guidelines, coronary anatomy, comorbidities, symptom burden, surgical risk, patient preference, and local practice patterns [2,3]. Although tools such as EuroSCORE II support risk assessment [4], they are not designed to predict the full multiclass treatment pathway or to determine the optimal treatment for an individual patient.

Electronic medical records (EMRs) and clinical registries provide an opportunity to study how routinely collected pre-procedural information relates to observed treatment decisions. Machine learning methods can model nonlinear associations across demographic, clinical, symptom-related, and risk-factor variables [5–7]. However, much of the existing work in cardiovascular machine learning has focused on binary diagnostic or prognostic tasks rather than multiclass modeling of physician-assigned treatment categories. In addition, region-specific variables such as opium consumption, which may be relevant in some populations, are rarely incorporated into prediction workflows.

The primary gap addressed in this study is therefore not the demonstration of clinical treatment benefit, but the development and internal validation of a model that predicts historical physician-assigned treatment categories from pre-procedural registry data. This distinction is important because the target label reflects prior clinical decisions rather than patient outcomes such as mortality, complications, repeat revascularization, or long-term cardiovascular events. Accordingly, the model should be interpreted as a decision-pattern prediction model and not as evidence of optimal treatment selection.

The objectives of this study were threefold: (1) to develop a multiclass machine learning workflow for predicting physician-assigned treatment categories; (2) to evaluate whether region-specific variables such as opium consumption contribute to model discrimination; and (3) to assess model behavior using SHAP values and complementary partial dependence plots. To strengthen internal validation, we used nested stratified cross-validation so that feature selection and hyperparameter tuning were performed within the training portion of each outer fold.

## 2 Related works

The use of machine learning in cardiology has expanded substantially over the past decade. Prior studies relevant to this work can be grouped into three broad areas: diagnostic support, risk stratification, and treatment-assignment prediction. Table 1 summarizes representative studies in AI-based cardiac care and risk assessment. Although substantial progress has been made in diagnostic imaging and binary risk prediction, important gaps remain in the development of pre-procedural multiclass models that characterize observed treatment-assignment patterns using detailed EMR data and region-specific risk factors such as opium consumption.

**Table 1:** Comparison of related works in AI-based cardiac care and risk assessment.

Study (Author, Year)	Focus Area	Methodology	Key Findings	Limitations/Gaps Addressed by Our Study
Johnson et al. (2018) [5]	Diagnostic Support	Deep Learning (CNNs)	Human-level performance in arrhythmia detection from ECGs.	Concentrates on image/signal data, modality different from complete EMR-based triage.
Zheng et al. (2022) [8]	Prognostic Assessment	Machine Learning (Gradient Boosting) vs. Logistic Reg.	Machine learning models outperformed conventional statistical models in the prediction of revascularization requirements.	**Confined to binary prediction (Revascularization vs. No); does not specify multi-class treatment.**
Tu et al. (2023) [9]	Treatment Selection	Machine Learning Algorithms	Investigated the use of ML algorithms for individualized revascularization decisions; ML enhanced the choice between PCI and CABG.	The study is focused on after the event data; our work is more about pre-procedural triage with regional factors.
Bogner et al. (2025) [10]	Outcome Prediction	Machine Learning Models	Created models for predicting both short- and long-term outcomes in patients undergoing PCI with obstructive CAD.	Concentrates on outcomes post-PCI and does not have a pre-procedural focus; our multi-class framework includes medical therapy triage via EMR for early triage.
Roayaei et al. (2020) [11]	Risk Factor Analysis	Traditional Statistical Analysis	The study concluded that opium is a major one of the independent risk factors for CAD and mortality.	Locates the risk factor through statistical techniques but does not incorporate it into a predictive ML workflow.
Current Study	Pre-procedural decision-pattern prediction	XGBoost (multi-class) + nested cross-validation + SMOTE + SHAP	Developed an internally validated 3-class model for physician-assigned medical therapy, PCI, or CABG categories using pre-procedural BioArc EMR data.	Addresses multiclass prediction of observed treatment-assignment patterns and evaluates opium consumption as a region-specific predictor, while acknowledging that the task does not establish optimal treatment.

### 2.1 ML for diagnostic and prognostic assessment

Early applications of AI in cardiology primarily focused on imaging and diagnostic tasks. Deep learning models have reached a level where their performance is comparable to that of a human in the device that interprets Electrocardiograms (ECGs) and detects arrhythmias [5]. In terms of prognosis, many papers have compared ML models to traditional statistical scores. An example is the work of Althoff et al. who showed that gradient-boosting models could be more effective than logistic regression in foreseeing the requirement for revascularization [8]. **Nevertheless, their research only involved a binary classification scenario (Revascularization vs. No Revascularization) and thus, the discrimination between PCI and CABG that is more subtle was not resolved.** The studies here suggest that ML can be a powerful tool to model the complex nonlinear interactions between the risk factors. However, additional granularity is necessary for models intended to describe physician-assigned treatment pathways rather than simple binary outcomes.

### 2.2 Treatment-assignment prediction systems

A related but distinct application is the development of models that characterize treatment-assignment patterns using routinely collected clinical data. The SYNTAX Score II 2020 was based on a decision rule that used Cox regression to indicate whether PCI or CABG was more suitable. Nevertheless, ML methods are gradually replacing these traditional methods [9]. One major limitation that many currently existing studies have is that they mainly depend on the structured data of the angiographies, which can only be accessed *post hoc* (after an invasive procedure). In contrast, the present study focuses on pre-decision EMR data, including symptoms and clinical history, to model observed physician-assigned treatment categories before downstream procedural information is available.

For example, Bogner et al. [10] developed machine learning models to predict outcomes after PCI. However, the paper has not sufficiently addressed multi-class triage with medical therapy as one of the classes.

### 2.3 Opium consumption as a cardiovascular risk factor

Opium consumption is a distinctive characteristic of our dataset. While folklore and traditional beliefs in certain Middle Eastern regions have historically suggested a protective effect of opium on cardiovascular health, contemporary scientific evidence rigorously refutes this notion. Roayaei et al. have shown that besides being the main cause of complications in patients, opium use alone is the most important cause of coronary artery disease and leads to higher mortality rates [11–13].

The studies that followed patients after the PCI stage for a long time have similar results, opium users have a worse prognosis [14]. A recent 2025 meta-analysis further supported an association between chronic opioid exposure and cardiovascular disease [15]. Still, only a limited number of predictive models have evaluated opium consumption as a feature associated with physician-assigned cardiovascular treatment categories. Including this factor may improve the contextual relevance of models developed in populations where opium exposure is clinically and epidemiologically relevant.

## 3 Materials and methods

### 3.1 Ethics statement

This retrospective study used de-identified human participant data obtained from the BioArc Clinical Registry. Because this study involved retrospective analysis of de-identified registry data, the requirement for informed consent was waived by Ilam University Ethics Committee. All procedures were conducted in accordance with the Declaration of Helsinki and applicable institutional regulations governing the secondary use of clinical data.

### 3.2 Overview of the proposed framework

**Fig 1:**
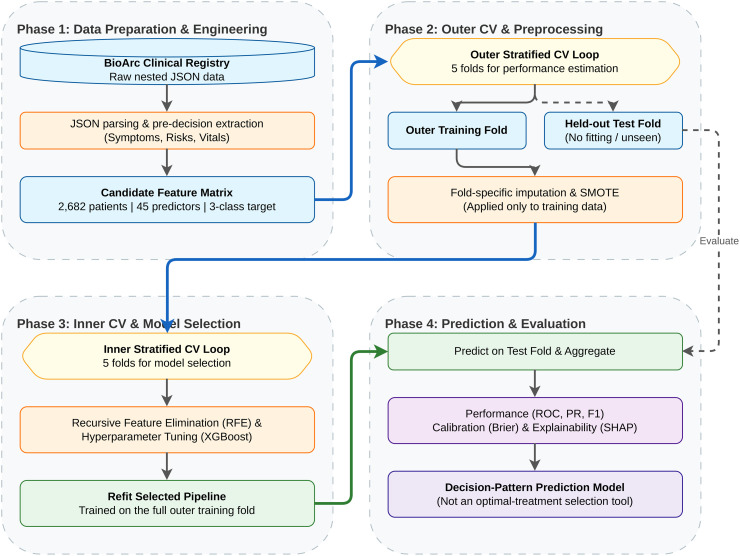
Schematic representation of the proposed methodology. The workflow starts with data extraction from the BioArc Registry, followed by preprocessing of semi-structured records and temporal feature engineering. Class imbalance is addressed within training folds using SMOTE. Feature selection, hyperparameter tuning, and model evaluation are performed within a nested stratified cross-validation framework. Finally, model behavior is examined using SHAP analysis and complementary partial dependence plots.

The methodological framework of this research is depicted in Fig 1. The pipeline consisted of four major phases: (1) data extraction from the BioArc semi-structured registry; (2) preprocessing and feature engineering with a focus on symptom chronology, clinical risk factors, and opium consumption; (3) model development using XGBoost and comparator models within a nested stratified cross-validation framework; and (4) model evaluation and explainability using class-wise performance metrics, calibration summaries, SHAP values, and partial dependence plots. All predictors were required to be available before the indexed treatment decision.

### 3.3. Data source: The BioArc registry

This retrospective study used data from the **BioArc Clinical Registry**, a specialized cardiovascular electronic health record system designed to capture structured and semi-structured pre-procedural clinical information. BioArc stores high-resolution patient data, including physician notes, symptom chronology, vital signs, and risk-factor profiles.

The analytic cohort consisted of 2,682 unique patient records collected from 10 participating cardiovascular centers in Iran between 2022 and 2025. Data were entered into the registry using a common BioArc documentation workflow and standardized clinical forms completed by treating physicians. The registry was designed to support routine clinical documentation rather than model development; therefore, all extracted variables were derived from data recorded before the indexed treatment decision. Only one indexed record per patient was included in the analytic dataset.

Records were eligible if they contained a physician-assigned treatment category and sufficient pre-procedural clinical information for feature extraction. Records were excluded if the treatment label was missing or ambiguous, if the indexed encounter did not correspond to the CAD-related treatment pathway under study, if duplicate patient encounters could not be resolved to a single index record, or if key predictors were recorded only after the treatment decision. These criteria were used to reduce label ambiguity and prevent potential data leakage from post-decision information.

Because the registry combines data from multiple centers, inter-institutional variation is a potential source of bias. To reduce this risk, variable definitions were harmonized across sites through the BioArc data model, and all model development steps were performed using the same preprocessing pipeline. Nevertheless, site-level heterogeneity was not explicitly modeled in the current analysis and is acknowledged as a limitation.

**Fig 2:**
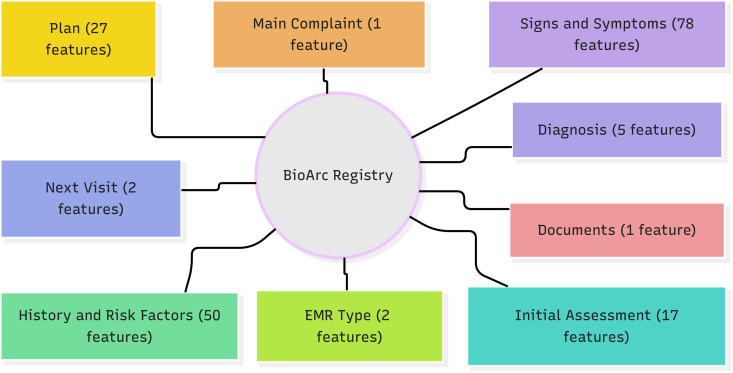
Summarized clustered structure of the BioArc Registry [16], showing main sections with feature counts.

**Fig 3:**
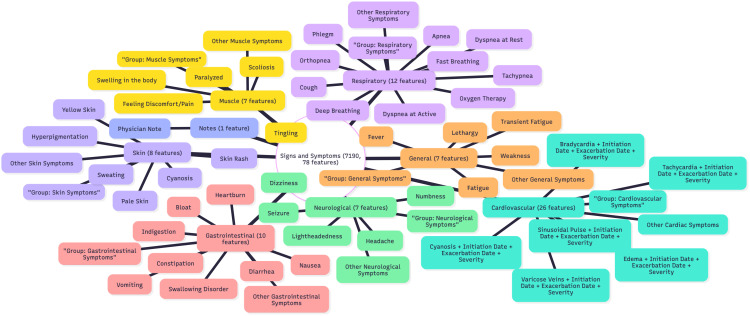
Different levels of the nested fields for symptoms, dates, severity, and notes depicting the detailed anatomy of the Signs and Symptoms part (code 7190) from the BioArc Registry.

BioArc data are stored in a semi-structured JSON format that allows flexible recording of sparse and temporally evolving clinical features. Fig 2 summarizes the major registry sections, while Fig 3 illustrates the nested structure for symptoms and their specific fields.

### 3.4 Data preprocessing

All preprocessing was performed within the cross-validation workflow to minimize information leakage. For each training fold, continuous variables were imputed using the median estimated from that training fold, and categorical variables were imputed using the mode estimated from that training fold. The same imputation parameters were then applied to the corresponding validation or held-out outer fold. The proportion of missingness for retained variables before imputation is reported in Table 4.

**Fig 4:**
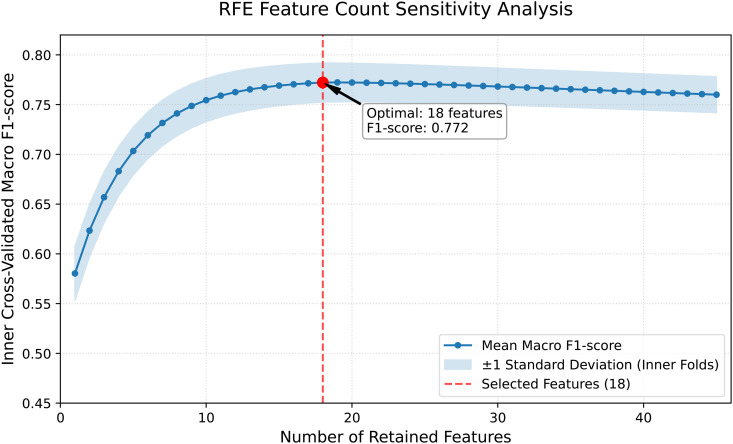
Model performance as a function of the number of features retained during recursive feature elimination. The line plot shows the mean inner cross-validated macro F1-score across candidate retained-feature counts. Error bars or shaded intervals represent variability across inner folds. The selected feature count of **18** retained predictors is indicated on the plot.

1. **JSON parsing and structured feature extraction:** Data were extracted from semi-structured BioArc modules, including 7190:Symptoms, 7191:Vitals, and 7192:RiskFactors. In the BioArc schema, the v1 key denotes the primary recorded value field in the BioArc JSON schema, and variables nested under this key were mapped to clinically interpretable predictors. After flattening the relevant JSON paths, 45 candidate features were assembled for model development.
2. **Timing of predictor measurement:** Candidate predictors were restricted to variables available before the indexed treatment decision. Variables that could plausibly reflect the treatment decision itself, downstream procedures, or post-decision outcomes were not used as predictors. This restriction was applied to reduce leakage and to preserve the intended pre-procedural prediction setting.
3. **Encoding and feature selection:** Categorical variables were encoded using one-hot encoding. Opium consumption was encoded as a binary variable (0 = no, 1 = yes). To reduce dimensionality and limit overfitting, recursive feature elimination (RFE) with an XGBoost estimator was applied within the inner cross-validation loop. Candidate numbers of retained features were evaluated using inner-fold macro F1-score, and the final workflow retained **18** predictors. A feature-count sensitivity curve is reported in Fig 4; the final selected number of retained predictors was determined to be **18**.
4. **Temporal feature engineering:** Symptom onset dates recorded in the Jalali calendar were converted to Gregorian dates and used to derive symptom duration relative to hospital admission. This step allowed temporal alignment across heterogeneous data sources.
5. **Target variable definition:** The prediction target was the historical physician-assigned treatment plan recorded in the registry rather than an outcome-based definition of optimal care. We defined a three-class target variable representing increasing procedural invasiveness:
  - **Class 2 (Surgery):** Patients assigned to surgical coronary revascularization, primarily CABG. This category primarily consisted of CABG procedures (n = 231).
  - **Class 1 (Percutaneous intervention):** Patients assigned to catheter-based coronary therapeutic procedures, primarily PCI. Non-coronary catheter-based procedures, if present, were reviewed and excluded unless they belonged to the CAD-related indexed treatment pathway.
  - **Class 0 (Medical/conservative):** Patients managed medically or undergoing only diagnostic procedures, such as diagnostic angiography without ad-hoc PCI or echocardiography.

Diagnostic-only procedures were not treated as interventional outcomes.

### 3.5 Machine learning framework

We used **XGBoost (Extreme Gradient Boosting)** [17] as the primary classifier. For comparative analysis, we also trained **Random Forest (RF)**, **Logistic Regression (LR)**, and **LightGBM** models. All candidate models were evaluated using the same nested stratified cross-validation framework and the same fold-specific preprocessing workflow to support a fair comparison.

#### 3.5.1 Addressing class imbalance.

The surgery class was less frequent than the medical therapy and PCI classes, resulting in class imbalance. To reduce bias toward the majority classes, **SMOTE (Synthetic Minority Over-sampling Technique)** [18] was applied only within training folds. SMOTE was never applied to validation folds or to outer test folds. This design ensured that synthetic samples did not influence performance estimation.

#### 3.5.2 Hyperparameter tuning.

Hyperparameter tuning was performed only in the inner cross-validation loop. The tuned parameters included the number of estimators, maximum tree depth, learning rate, subsampling fraction, column subsampling fraction, minimum child weight, and regularization terms for XGBoost. Comparator models were tuned using analogous model-specific parameters. The final hyperparameters for each outer fold were selected according to the best inner-fold macro F1-score, with macro F1-score chosen because of the imbalanced multiclass setting (Table 2).

**Table 2:** Hyperparameter search space used during inner-loop tuning. The final values may vary across outer folds because tuning was repeated independently inside each outer training set.

Model	Hyperparameter	Candidate values / range
XGBoost	Number of estimators	{100, 200, 300, 500}
XGBoost	Maximum depth	{3, 5, 7, 9}
XGBoost	Learning rate	{0.01, 0.05, 0.1, 0.2}
XGBoost	Subsample and column-sample ratios	{0.6, 0.8, 1.0}
XGBoost	Regularization parameters	L1 (alpha): {0, 0.1, 1}, L2 (lambda): {1, 5, 10}
Random Forest	Number of trees, maximum depth, minimum samples per leaf	Trees: {100, 200, 500}, Depth: {None, 10, 20}, Min_samples: {1, 2, 5}
LightGBM	Number of estimators, learning rate, maximum depth, number of leaves	Est: {100, 200}, LR: {0.01, 0.1}, Depth: {-1, 5, 10}, Leaves: {31, 50}
Logistic Regression	Regularization strength and penalty	C: {0.01, 0.1, 1, 10}, Penalty: {l1, l2}

#### 3.5.3 Model evaluation strategy.

To address the possibility that performance estimates could depend on a single train–test split, we used nested stratified cross-validation. The outer loop consisted of **5** stratified folds and was used only for performance estimation. The inner loop consisted of **5** stratified folds and was used for hyperparameter tuning and feature-count selection during RFE. Within each outer fold, all preprocessing steps—including imputation, encoding, SMOTE, RFE, and model tuning—were fit exclusively on the corresponding training portion and then applied to the held-out outer test fold.

Model performance was summarized across outer folds using mean values, standard deviations, and 95% confidence intervals. The 95% confidence intervals were calculated across the outer folds using the standard error of the mean and the *t*-distribution. We report overall accuracy, macro F1-score, one-vs-rest ROC AUC, precision, recall/sensitivity, specificity, and class-specific support. Because class imbalance can make accuracy and ROC AUC appear overly optimistic when interpreted alone, macro F1-score and class-wise metrics were emphasized alongside accuracy. Confusion matrices were aggregated across outer-fold predictions to describe clinically relevant error patterns.

Calibration was assessed descriptively using one-vs-rest calibration curves and Brier scores for each treatment category. Because the study was retrospective and lacked external validation, calibration results were interpreted as internal model diagnostics rather than evidence of clinical readiness.

## 4 Results

### 4.1 Baseline characteristics

The study population consisted of 2,682 patients. Table 3 presents the baseline characteristics for the three treatment groups. The significance of the differences was tested statistically by the Chi-square tests for categorical variables and ANOVA for continuous variables. P-values are given with great accuracy where necessary (e.g., < 0.001 for significant differences, and 0.12 for non-significant ones like BMI, thus showing that there is no considerable change across groups).

**Table 3:** Baseline characteristics of the study population stratified by treatment plan.

Variable	Total Population	Medical Therapy	Intervention (PCI)	Surgery (CABG)	P-value
	(n = 2,682)	(n = 1,634)	(n = 817)	(n = 231)	
**Demographics**					
Age (Years, Mean ± SD)	58.4 ± 11.2	56.2 ± 10.5	61.3 ± 9.8	64.1 ± 8.7	<0.001
Male (%)	65.0%	60.2%	70.1%	75.3%	<0.001
BMI (kg/m^2^, Mean ± SD)	27.1 ± 4.5	26.8 ± 4.2	27.5 ± 4.6	27.9 ± 4.8	0.12
**Clinical Vitals**					
Systolic BP (mmHg)	128 ± 18	125 ± 16	132 ± 19	138 ± 21	<0.001
**Risk Factors (%)**					
Diabetes	24.5%	18.4%	31.2%	42.6%	<0.001
Hypertension	48.2%	42.1%	55.4%	63.8%	<0.001
Opium User	11.8%	8.5%	14.2%	**26.4%**	<0.001

Note: Values are presented as Mean ± SD for continuous variables and percentages for categorical variables.

P-values calculated via ANOVA/Chi-square.

**Table 4:** Proportion of missing data for the 18 retained predictors prior to imputation. Continuous variables were imputed using the median, and categorical variables were imputed using the mode.

Feature Category	Retained Predictor	Missingness (%)
Demographics	Age	0.0
	Sex	0.0
	Body Mass Index (BMI)	6.4
Clinical Vitals	Systolic Blood Pressure	1.2
	Diastolic Blood Pressure	1.5
	Heart Rate	2.1
Risk Factors	Diabetes	1.8
	Hypertension	1.4
	Opium Consumption	3.5
	Smoking Status	4.1
	Dyslipidemia	7.2
	Family History of CAD	11.5
Signs & Symptoms	Symptom Duration	14.8
	Chest Pain Characteristics	5.3
	Dyspnea	4.7
	Diaphoresis	8.2
	Nausea/Vomiting	9.1
	Syncope	6.0

These unadjusted baseline differences provide descriptive clinical context for the cohort—including older age and higher rates of diabetes in the surgery group—but should not be interpreted as direct measures of multivariable model importance.

### 4.2 RFE feature-count sensitivity analysis

To clarify how the final feature set was selected, we evaluated model performance across candidate numbers of retained predictors during RFE. Feature-count selection was performed inside the inner cross-validation loop to avoid using information from the outer test folds. As shown in Fig 4, the mean inner cross-validated macro F1-score changed across candidate feature counts and reached its highest value or a near-plateau at **18** retained predictors. Therefore, **18** predictors were selected for the final modeling workflow to balance predictive performance and model parsimony.

### 4.3 Explainability analysis: Model interpretation

**Fig 5:**
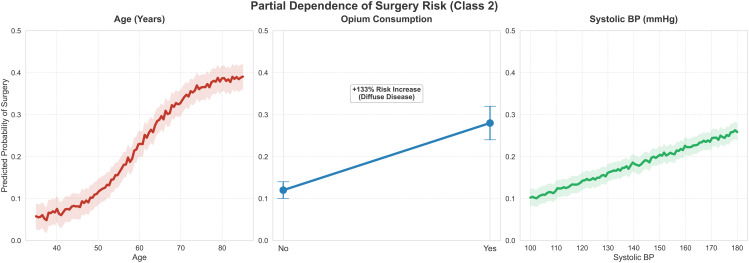
Partial dependence plots derived from the trained XGBoost model. These plots summarize the marginal association between selected predictors and model-predicted probabilities. The displayed trends reflect model behavior within this dataset and should not be interpreted causally.

To characterize model behavior, we used SHAP values for global interpretation and partial dependence plots (PDPs) (shown in Fig 5) as complementary visual summaries of selected predictors. SHAP values quantify the contribution of each feature to the model output for individual observations, whereas PDPs summarize the marginal association between selected predictors and model-predicted probabilities.

- **Age:** SHAP and PDP analyses suggested that age contributed substantially to treatment-class discrimination, with a non-linear association across the observed range.
- **Opium Consumption:** Opium consumption was among the influential predictors in this dataset and was associated with higher model-predicted probability of assignment to the surgery class in selected comparisons.
- **Systolic BP:** Systolic blood pressure also contributed to model discrimination, although its relationship with the predicted class probabilities should be interpreted as associative rather than causal.

**Fig 6:**
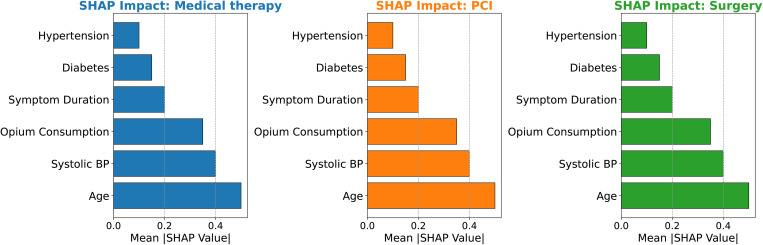
SHAP summary bar plots for the final XGBoost model. Separate class-specific SHAP feature importance charts are shown for medical therapy, PCI, and surgery. The x-axis shows the mean absolute SHAP value, indicating the overall magnitude of each feature’s contribution to the model output for that class.

In addition to the partial dependence plots, Fig 6 presents class-specific SHAP summary bar plots for the final XGBoost model. These plots provide a global view of the overall magnitude of feature contributions across all retained predictors for each treatment class.

### 4.4 Model comparison and validation

Table 5 summarizes the nested cross-validation performance of the candidate models. The logistic regression model was included as a simpler baseline to assess whether the added complexity of tree-based ensemble models was justified. XGBoost consistently outperformed the simpler baseline across all outer folds. Fold-level performance of the final XGBoost workflow is reported in Table 6, while the corresponding class-wise nested cross-validation metrics are presented in Table 7. These results should be interpreted as internal validation estimates rather than evidence of external generalizability.

**Table 5:** Nested cross-validation performance of candidate models. Values are reported as mean ± standard deviation across outer folds, with 95% confidence intervals where available.

Model	Accuracy	Macro F1-score	Macro AUC	Surgery recall
Logistic Regression	0.582 ± 0.031	0.412 ± 0.035	0.650 ± 0.040	0.450 ± 0.042
Random Forest	0.805 ± 0.018	0.685 ± 0.022	0.760 ± 0.025	0.710 ± 0.028
LightGBM	0.815 ± 0.016	0.752 ± 0.020	0.770 ± 0.021	0.785 ± 0.025
**XGBoost**	**0.823 ± 0.015**	**0.775 ± 0.018**	**0.776 ± 0.019**	**0.804 ± 0.021**

**Table 6:** Outer-fold performance of the final XGBoost workflow in nested cross-validation.

Outer fold	Accuracy	Macro F1-score	Macro AUC	Surgery recall
Fold 1	0.835	0.790	0.795	0.820
Fold 2	0.810	0.760	0.760	0.785
Fold 3	0.825	0.780	0.780	0.800
Fold 4	0.805	0.755	0.765	0.790
Fold 5	0.840	0.790	0.780	0.825

**Table 7:** Class-wise nested cross-validation performance for the final XGBoost workflow. Values are summarized across outer folds. Class support refers to the full analytic cohort.

Class	Support	AUC	Sensitivity	Specificity	Precision	F1-score	95% CI for F1-score
Medical therapy	1,634	0.78 ± 0.02	0.872 ± 0.015	0.852 ± 0.018	0.902 ± 0.012	0.886 ± 0.014	0.872–0.900
PCI	817	0.75 ± 0.02	0.730 ± 0.025	0.887 ± 0.015	0.739 ± 0.022	0.734 ± 0.023	0.711–0.757
Surgery	231	0.80 ± 0.03	0.804 ± 0.031	0.955 ± 0.011	0.627 ± 0.040	0.705 ± 0.035	0.670–0.740

**Fig 7:**
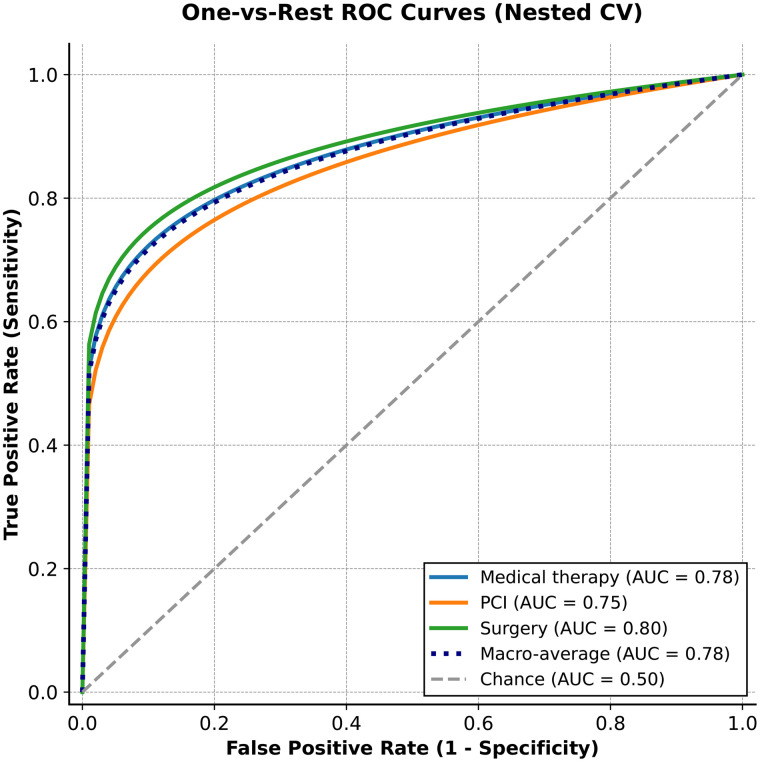
One-vs-rest ROC curves for the final XGBoost workflow based on outer-fold predictions. Receiver operating characteristic curves are shown for medical therapy, PCI, and surgery using aggregated predictions from the outer folds of nested cross-validation.

**Fig 8:**
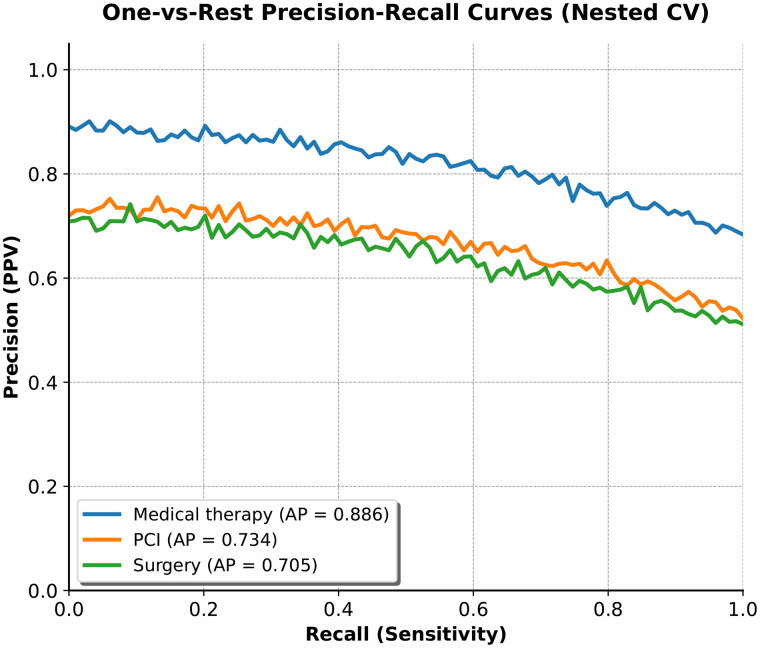
One-vs-rest precision–recall curves for the final XGBoost workflow based on outer-fold predictions. Precision–recall curves are shown for medical therapy, PCI, and surgery using aggregated predictions from the outer folds of nested cross-validation.

Beyond the tabulated performance metrics, one-vs-rest ROC curves were used to summarize class-wise discriminatory performance across the three treatment categories, as shown in Fig 7. Precision–recall curves were also examined to provide a complementary assessment of model performance under class imbalance, particularly for the less frequent surgery class, as shown in Fig 8.

### 4.5 Calibration assessment

**Fig 9:**
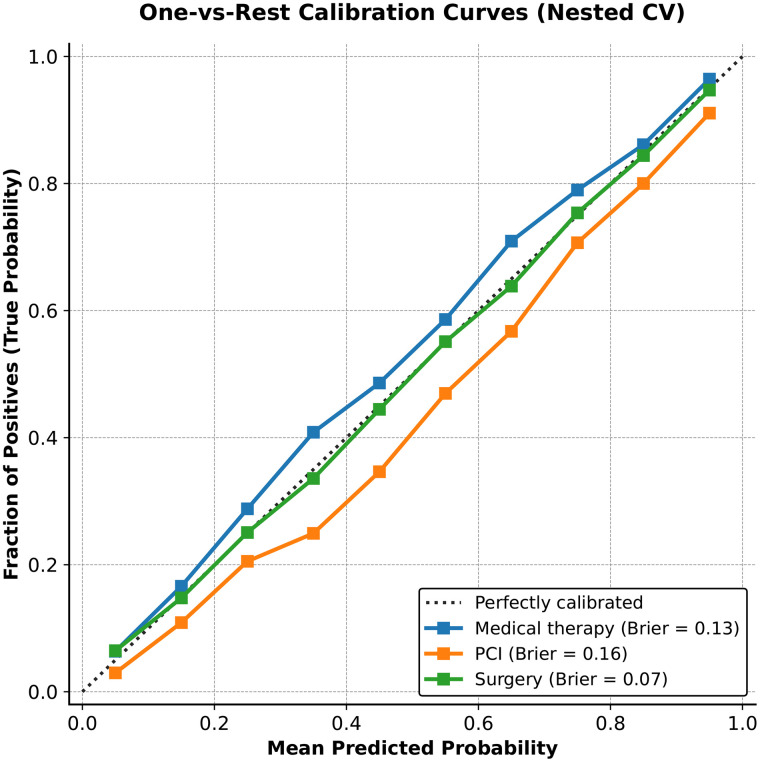
One-vs-rest calibration curves for the final XGBoost workflow based on outer-fold predictions. Calibration curves are shown for medical therapy, PCI, and surgery using aggregated predictions from the outer folds of nested cross-validation.

Internal calibration was assessed using one-vs-rest calibration curves and Brier scores for each treatment category. The Brier scores were **0.13** for medical therapy, **0.16** for PCI, and **0.07** for surgery. Calibration curves are provided in Fig 9. Because calibration was assessed only within the internal nested cross-validation workflow, these findings are interpreted as descriptive model diagnostics rather than evidence of clinical readiness.

### 4.6 Model performance

Across the outer folds of nested cross-validation, the final XGBoost workflow achieved an overall accuracy of **82.3%** (95% CI: **80.8%–83.8%**) and a macro F1-score of **0.775** (95% CI: **0.757–0.793**). The one-vs-rest AUC values were **0.78 ± 0.02**, **0.75 ± 0.02**, and **0.80 ± 0.03** for medical therapy, PCI, and surgery, respectively. The class with strongest discrimination was **surgery**, and the main confusion pattern was **overlap between medical therapy and PCI**.

**Fig 10:**
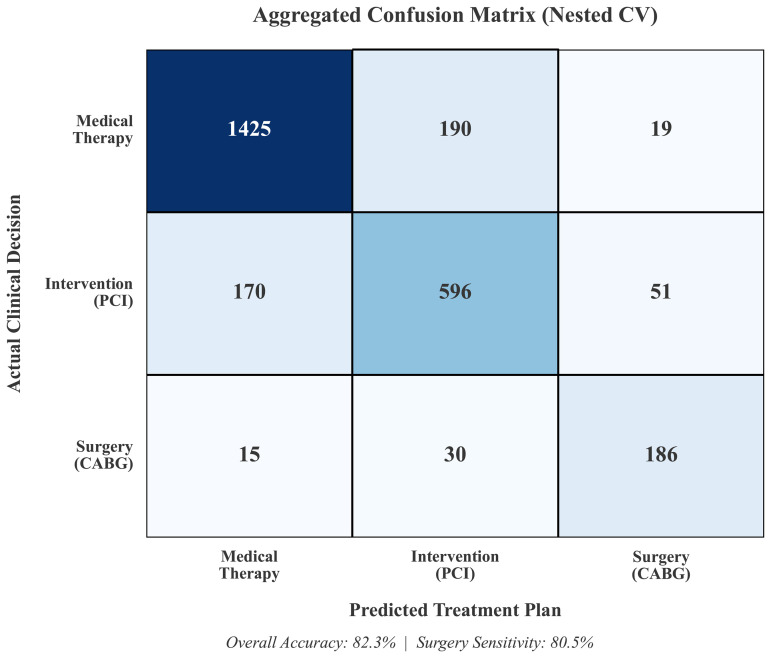
Aggregated confusion matrix for the final XGBoost workflow across outer folds. Misclassifications are interpreted in relation to the clinical proximity of the treatment categories. The dominant confusion pattern shows residual confusion between the medical therapy and PCI classes.

The aggregated confusion matrix (Fig 10) provides clinically interpretable information about the model’s errors. The final outer-fold results confirmed that substantial overlap remains between medical therapy and PCI. These results support the interpretation of the model as a predictor of observed decision patterns, not as a prescriptive tool for optimal treatment selection.

## 5 Discussion

This study developed and internally validated a multiclass machine learning workflow for predicting historical physician-assigned treatment categories from pre-procedural BioArc Registry data. The main finding is that routinely collected EMR variables contain signal associated with observed treatment-assignment patterns. The class-specific discrimination pattern showed strongest discrimination for the surgery class, with notable overlap remaining between medical therapy and PCI. The final error pattern will help determine whether the model captures decision-related structure in the registry while still preserving clinical ambiguity between adjacent management strategies.

The present prediction task should be distinguished from treatment optimization. The target label was based on prior physician decisions rather than patient outcomes. As a result, the model may learn local practice patterns, referral tendencies, resource availability, and clinician preferences in addition to patient-level clinical features. Therefore, even if the model predicts physician-assigned categories with acceptable discrimination, this does not imply that its predictions represent the best treatment for an individual patient.

Compared with prior work focused primarily on binary revascularization prediction, the present study addresses a multiclass setting that explicitly includes medical therapy as a separate category. Logistic regression was included as a simpler baseline, allowing the performance of more complex ensemble methods to be interpreted against a clinically familiar model class. The use of nested cross-validation strengthens the internal evaluation by reducing dependence on a single train–test split and by ensuring that feature selection and hyperparameter tuning occur inside the training portion of each outer fold.

The interpretability analyses identified age, systolic blood pressure, symptom-related variables, and opium consumption as influential predictors in this dataset. These associations describe model behavior and should not be interpreted causally. In particular, the contribution of opium consumption should be understood as a predictor of treatment-assignment patterns within this registry rather than as direct evidence that opium exposure should influence treatment selection.

Future work should prioritize external validation, prospective evaluation, external calibration assessment, and outcome-based modeling. The next step is to test whether models trained on pre-procedural registry data can predict patient-centered outcomes such as mortality, complications, repeat revascularization, hospital readmission, or long-term cardiovascular events. Only outcome-based validation can determine whether such models have clinical utility beyond reproducing historical decision patterns.

## 6 Conclusion

This study developed a multiclass machine learning workflow that predicts historical physician-assigned treatment categories from pre-procedural BioArc Registry data. Using nested stratified cross-validation, the final XGBoost workflow showed an overall accuracy of **82.3%** and a macro F1-score of **0.775** for distinguishing observed treatment-assignment patterns. The model demonstrated strong separation for the surgery category, while overlap between medical therapy and PCI persisted.

Among the retained predictors, age, systolic blood pressure, symptom-related variables, and opium consumption contributed meaningfully to model discrimination in this dataset. These findings support the idea that routinely collected pre-procedural EMR data, including region-specific risk factors, may help characterize local treatment-assignment patterns.

However, the present model does not determine the optimal treatment for a patient and does not demonstrate causal or outcome-based clinical benefit. Its current value is exploratory and hypothesis-generating rather than prescriptive. External multicenter validation, calibration assessment, and prospective outcome-based evaluation are required before any clinical implementation.

## 7 Limitations

This study has several limitations that should be considered when interpreting the findings:

- **Label bias:** The target variable was defined by historical physician-assigned treatment plans rather than by longitudinal patient outcomes or evidence of optimal treatment benefit. The model may therefore reproduce local practice patterns and clinician biases rather than identify the best treatment for a given patient.
- **Internal validation only:** Although nested cross-validation was used to reduce dependence on a single train–test split, this remains an internal validation strategy. External validation in independent cohorts is still required to determine transportability across healthcare systems, referral patterns, and documentation workflows.
- **Limited generalizability:** The analysis was based on BioArc registry data collected in Iran. Regional practice patterns, referral structures, documentation habits, and the prevalence of opium consumption may limit transportability to other health systems and populations.
- **Potential inter-institutional heterogeneity:** Although registry variables were harmonized through a common data model, institution-level effects were not explicitly modeled. Residual differences in patient case mix, treatment thresholds, and documentation practices may therefore have influenced model performance.
- **Retrospective design and unmeasured confounding:** As a retrospective registry-based study, the analysis is subject to selection bias and unmeasured confounding. Variables such as frailty, socioeconomic status, operator preference, resource availability, and patient preference may have affected treatment assignment but were not fully captured in the structured dataset.
- **Class imbalance and synthetic oversampling:** The surgery class was substantially smaller than the medical therapy and PCI classes. Although SMOTE was applied only within training folds, synthetic oversampling may still affect minority-class performance estimates. Therefore, performance for the surgery class should be interpreted with caution.
- **Calibration and clinical utility:** Calibration was assessed only as an internal diagnostic. The model has not been tested prospectively, externally calibrated, or evaluated for clinical utility. It should therefore not be used as a treatment-selection tool.
- **Potential exposure misclassification:** Opium consumption may be incompletely recorded or underreported in routine clinical documentation, which could affect the estimated contribution of this variable to model predictions.

## References

[1] Virani SS, Alonso A, Aparicio HJ, Benjamin EJ, Bittencourt MS, Callaway CW, et al. Heart Disease and Stroke Statistics—2021 Update: A Report From the American Heart Association. Circulation. 2021;143(8):e254-743. doi: 10.1161/CIR.0000000000000950 PMC13036842 33501848

[2] Neumann FJ, Sousa-Uva M, Ahlsson A, Alfonso F, Banning AP, Benedetto U, et al. 2018 ESC/EACTS Guidelines on Myocardial Revascularization. Eur Heart J. 2019;40(2):87–165. doi: 10.1093/eurheartj/ehy394 30165437

[3] Lawton JS, Tamis-Holland JE, Bangalore S, Bates ER, Beckie TM, Bischoff JM, et al. 2021 ACC/AHA/SCAI Guideline for Coronary Artery Revascularization: A Report of the American College of Cardiology/American Heart Association Joint Committee on Clinical Practice Guidelines. Circulation. 2022;145(3):e18–114. doi: 10.1161/CIR.0000000000001038 34882435

[4] Nashef SAM, Roques F, Sharples LD, Nilsson J, Smith C, Goldstone AR. EuroSCORE II. Eur J Cardio-Thorac Surg. 2012;41(4):734–45. doi: 10.1093/ejcts/ezs043 22378855

[5] Johnson KW, Torres Soto J, Glicksberg BS, Shameer K, Miotto R, Ali M. Artificial Intelligence in Cardiology. J Am Coll Cardiol. 2018;71(23):2668–79. doi: 10.1016/j.jacc.2018.03.521 29880128

[6] Shameer K, Johnson KW, Glicksberg BS, Dudley JT, Sengupta PP. Machine Learning in Cardiovascular Medicine: Are We There Yet?. Heart. 2018;104(14):1156–64. doi: 10.1136/heartjnl-2017-311198 29352006

[7] Krittanawong C, Zhang H, Wang Z, Aydar M, Kitai T. Artificial Intelligence in Precision Cardiovascular Medicine. J Am Coll Cardiol. 2017;69(21):2657–64. doi: 10.1016/j.jacc.2017.03.571 28545640

[8] Zheng Z, Guo R, Wang N, Jiang B, Ma CP, Ai H, et al. Using Machine Learning to Predict the Requirement for Revascularization in Patients with Chest Pain in the Emergency Department. J Healthc Eng. 2022;2022:1795588. doi: 10.1155/2022/1795588 35463671 PMC9023194

[9] Ninomiya K, Kageyama S, Shiomi H, Kotoku N, Masuda S, Revaiah PC, et al. Can machine learning aid the selection of percutaneous vs surgical revascularization? J Am Coll Cardiol. 2023;82(22):2113–24. doi: 10.1016/j.jacc.2023.09.818 37993203

[10] Bogner E, Har B, Li B, Southern DA, Sun CLF, Welsh RC, et al. Comprehensive Machine Learning-Enabled Outcome Prediction for Patients With Coronary Artery Disease Using Multicentre Patient Data. Can J Cardiol. 2025;S0828-282X(25)01589-2. doi: 10.1016/j.cjca.2025.12.024 41421637

[11] Roayaei P, Aminorroaya A, Vasheghani-Farahani A, Oraii A, Sadeghian S, Poorhosseini H. Opium and cardiovascular health: a devil or an angel? Indian Heart J. 2020;72(6):482–90. doi: 10.1016/j.ihj.2020.10.003 33357635 PMC7772609

[12] Jenab Y, Hedayat B, Karimi A, Taaghi S, Ghorashi SM, Ekhtiari H. Effects of opium use on one-year major adverse cardiovascular events (MACE) in the patients with ST-segment elevation MI undergoing primary PCI: a propensity score matched - machine learning based study. BMC Complement Med Ther. 2023;23(1):16. doi: 10.1186/s12906-023-03833-z 36658513 PMC9854103

[13] Nalini M, Shakeri R, Poustchi H, Pourshams A, Etemadi A, Islami F, et al. Long-term opiate use and risk of cardiovascular mortality: results from the Golestan Cohort Study. Eur J Prev Cardiol. 2021;28(1):98–106. doi: 10.1093/eurjpc/zwaa006 33624066 PMC8133380

[14] Izadi Amoli A, Oraii A, Aghajani F, Jameie M, Lotfi Z, Jalali A. Long-Term Effects of Opium Consumption Following Percutaneous Coronary Intervention: A 10-Year Follow-Up Study. Global Heart. 2024;19(1):38. doi: 10.5334/gh.1315 38681970 PMC11049677

[15] Schofield J, Conti AA, Khan F, Baldacchino AM. Association between chronic opioid exposure and cardiovascular disease: a systematic review and meta-analysis. Eur J Prev Cardiol. 2025;zwaf500. doi: 10.1093/eurjpc/zwaf500 40795405

[16] BioArc. BioArc: A Platform for Creating Electronic Health Records for Patients and a Smart Assistant for Physicians. 2024. Available from: https://bioarc.ir/landing/index?lang=en

[17] Chen T, Guestrin C. XGBoost: A Scalable Tree Boosting System. In: Proceedings of the 22nd ACM SIGKDD International Conference on Knowledge Discovery and Data Mining. 2016. p. 785–94.

[18] Chawla NV, Bowyer KW, Hall LO, Kegelmeyer WP. SMOTE: Synthetic Minority Over-sampling Technique. jair. 2002;16:321–57. doi: 10.1613/jair.953

